# Phenotyping grapevine resistance to downy mildew: deep learning as a promising tool to assess sporulation and necrosis

**DOI:** 10.1186/s13007-024-01220-4

**Published:** 2024-06-13

**Authors:** Felicià Maviane Macia, Tyrone Possamai, Marie-Annick Dorne, Marie-Céline Lacombe, Eric Duchêne, Didier Merdinoglu, Nemo Peeters, David Rousseau, Sabine Wiedemann-Merdinoglu

**Affiliations:** 1 Laboratoire des Interactions Plantes Micro-organismes Environnement (LIPME), INRAE, CNRS, Université de Toulouse, Chem. de Borde Rouge, Castanet-Tolosan, France; 2 Université d’Angers, LARIS, INRAE, IRHS, Angers, France; 3 Santé de la Vigne et Qualité du Vin (SVQV), INRAE, Université de Strasbourg, Colmar, France

**Keywords:** Deep learning, *Vitis*, *Plasmopara viticola*, Grapevine resistance, High throughput phenotyping, OIV 452 descriptor

## Abstract

**Background:**

Downy mildew is a plant disease that affects all cultivated European grapevine varieties. The disease is caused by the oomycete *Plasmopara viticola*. The current strategy to control this threat relies on repeated applications of fungicides. The most eco-friendly and sustainable alternative solution would be to use bred-resistant varieties. During breeding programs, some wild *Vitis* species have been used as resistance sources to introduce resistance loci in *Vitis vinifera* varieties. To ensure the durability of resistance, resistant varieties are built on combinations of these loci, some of which are unfortunately already overcome by virulent pathogen strains. The development of a high-throughput machine learning phenotyping method is now essential for identifying new resistance loci.

**Results:**

Images of grapevine leaf discs infected with *P. viticola* were annotated with OIV 452–1 values, a standard scale, traditionally used by experts to assess resistance visually. This descriptor takes two variables into account the complete phenotype of the symptom: sporulation and necrosis. This annotated dataset was used to train neural networks. Various encoders were used to incorporate prior knowledge of the scale’s ordinality. The best results were obtained with the Swin transformer encoder which achieved an accuracy of 81.7%. Finally, from a biological point of view, the model described the studied trait and identified differences between genotypes in agreement with human observers, with an accuracy of 97% but at a high-throughput 650% faster than that of humans.

**Conclusion:**

This work provides a fast, full pipeline for image processing, including machine learning, to describe the symptoms of grapevine leaf discs infected with *P. viticola* using the OIV 452–1, a two-symptom standard scale that considers sporulation and necrosis. If symptoms are frequently assessed by visual observation, which is time-consuming, low-throughput, tedious, and expert dependent, the method developed sweeps away all these constraints. This method could be extended to other pathosystems studied on leaf discs where disease symptoms are scored with ordinal scales.

## Background

The European vine *Vitis vinifera L.* is one of the most economically important and widespread crops in the world. In 2020, vineyards spanning an estimated 7.3 million hectares were dedicated to producing wine, juice, table grapes and dry grapes [1]. Grapevines are affected by a variety of diseases [2], with one of the most damaging being downy mildew, caused by the oomycete *Plasmopara viticola* [3–5].The pathogen is endemic to North America and was first identified in Europe in the 1870s. Following this introduction, European populations spread to vineyards worldwide, significantly impacting viticulture [6]. Downy mildew is now a major disease in regions characterized by warm and wet conditions during the vegetative period. Globally, viticulture relies predominantly on grape varieties derived from the Eurasian species *Vitis vinifera* due to the high quality of its fruit. However, almost all *V. vinifera* varieties exhibit varying degrees of susceptibility to the pathogen [7–10]. The pathogen attacks all green parts of the vine, especially leaves, petioles, inflorescences, and bunches. In vineyards, symptoms appear on leaves as shiny and oily lesions with sporulation on their lower surface. Severely infected leaves can become necrotic, leading to defoliation. Currently, the main strategy for controlling this disease involves repeated fungicide applications, which occasionally result in the emergence of resistant strains [11]. A more environmentally friendly and sustainable approach is to cultivate new bred grape varieties with improved genetic resistance.

Long-lasting breeding programs have been conducted to introgress resistance from wild *Vitis* species into elite *Vitis vinifera* varieties [12, 13]. The resistance trait in wild species, hybrids, and varieties is determined by genetic loci named *Rpv* for “Resistance to *Plasmopara viticola*”. More than 30 *Rpv* genes have been identified (maul_ 2021). Some major *Rpv* loci introduced in breeding programs have already been disrupted by virulent *P. viticola* strains [14–16]. Consequently, breeders frequently employ the strategy of pyramiding multiple resistance loci within the same variety. This approach aims to enhance resistance levels, increase effectiveness against diverse strains, and ensure long-term durability. In this regard, an extended and accurate high-throughput phenotyping strategy is essential for identifying new *Rpv* loci.

Grapevine resistance is usually scored by visual ratings of macroscopic symptoms developed on leaves by using several variables, such as severity, incidence, and spore count or by using the OIV 452 descriptor [17]. The “Office International de la Vigne et du Vin” (OIV) proposes standardizing the assessment of numerous grapevine traits by using descriptors based on discrete scales ranging from 1 to 9. The OIV 452 descriptor has been frequently used to assess resistance on whole plants vineyards or under controlled laboratory conditions on leaf discs. The OIV 452 descriptor assesses resistance by considering both pathogen sporulation and the visible phenotypes of the plant’s response. The macroscopic response is visually characterized by necrosis, also known as the hypersensitive response (HR) or plant cell death (PCD). In leaves, most *Rpv* loci provide partial resistance, which is defined by varying degrees of sporulation and necrosis [16, 18–21]. Visual scoring requires trained experts but it is often time-consuming and tedious. Nevertheless, it remains the most widely used strategy for assessing downy mildew infection and grapevine resistance.

**Fig. 1:**
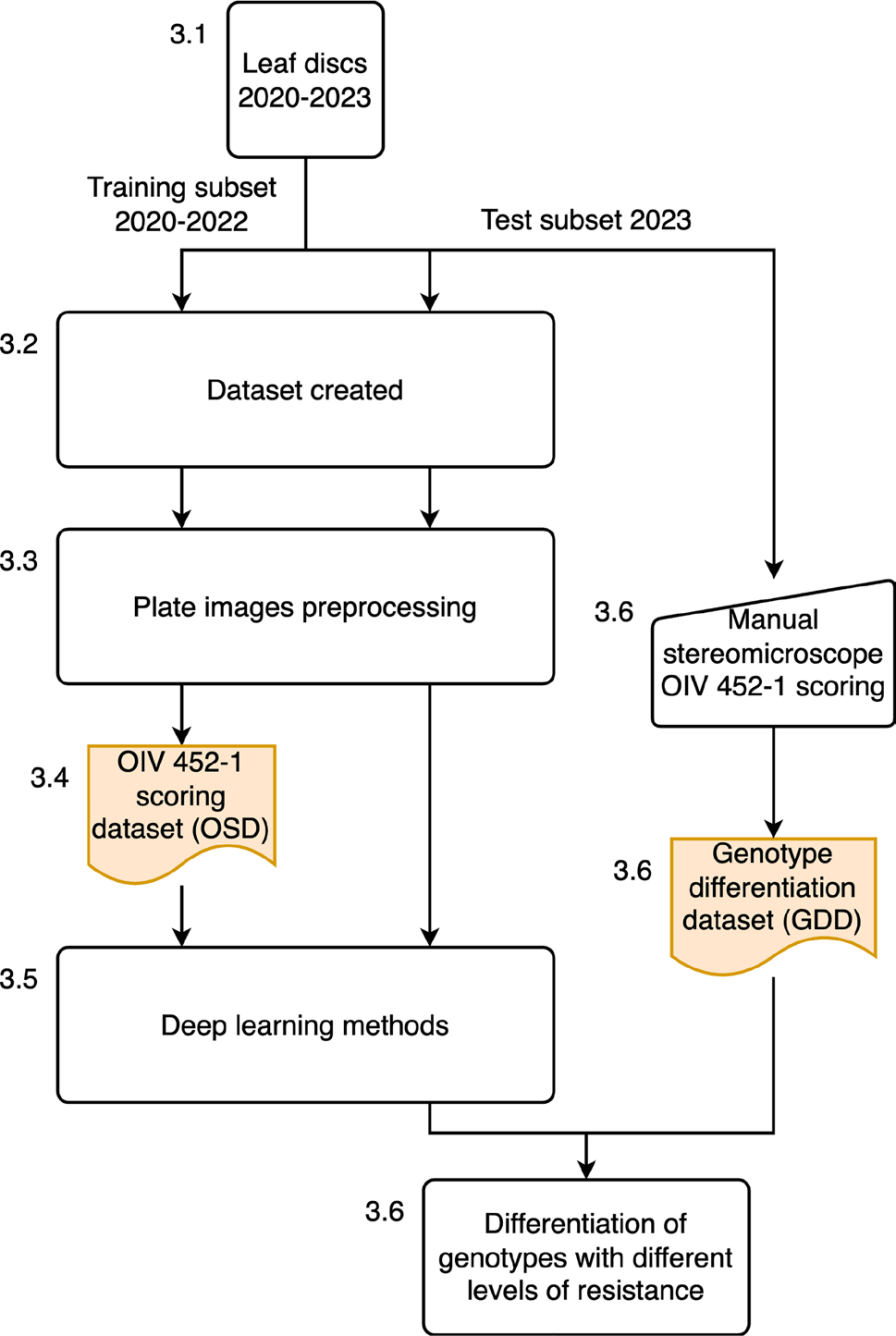
Global view of the proposed pipeline. The numbers in the figure and the caption refer to the subsections of the manuscript. Leaf discs infected with *P. viticola* between 2020 and 2022 (3.1) were imaged to create a dataset of plate images (3.2), which were preprocessed to extract leaf patches (3.3). A subset of these leaf patches was annotated with an OIV 452–1 score (3.4) and used to explore deep learning methods (3.5). Then, in 2023, leaf discs infected with *P. viticola* obtained following the same protocol were manually annotated with an OIV 452–1 score under a stereo-microscope (3.6), in parallel; OIV 452–1 scores were predicted using the model created in 3.5. The results of the manual annotation and model predictions were compared in 3.6

A fast and precise phenotyping scoring method, aligned with the OIV 452 standard (evaluating both *P. viticola* sporulation and host necrosis) yet tailored for high-throughput phenotyping, should be adopted to meet phenotyping requirements. In this work, as depicted in Fig. 1, we propose an automated method involving image acquisition and a deep learning pipeline based on annotated images of downy mildew symptoms on grapevine leaf discs.

Deep learning, a subset of machine learning, employs artificial neural networks with multiple hidden layers. One of its significant advantages lies in its ability to discern complex patterns in images. In theory, deep neural networks can match the performance of human experts, providing unbiased and consistent results.

To validate the model, we used a panel of genotypes known to display diverse phenotypes. The model’s image-based predictions were assessed by comparing them to data derived from human stereo-microscope observations, obtained using the OIV 452–1 scale, an extension of OIV 452 dedicated to leaf disc analysis.

## Related work

To the best of our knowledge, automatic phenotyping of the interaction between downy mildew and grapevine leaf discs has been described in only two articles [22, 23]. In the first study [22], the researchers sliced leaf discs into 506 segments. Then, they applied a shallow convolutional neural network to each segment to predict the presence or absence of sporangia. This analysis provided a comprehensive count of sporangia per leaf disc. In the second study [23], the authors applied fuzzy logic to isolate pixels showing sporulation in leaf disc images and used these data to compute a downy mildew severity score. Based on this score, the leaf discs were classified into three groups according to the severity of downy mildew sporulation: low (0–25% leaf disc coverage), medium (26 to 50% coverage) and high (more than 50% coverage). However, both articles assessed the interaction exclusively based on the sporulation variable, without considering a scale that also accounts for necrosis.

The authors of [24] used deep learning to detect the presence of *Erysiphe necator* hyphae on leaf discs. However, this study did not consider plant response to the pathogen. As more related work, the authors of [25] automatically classified the interaction between melon leaf discs and powdery mildew using deep learning. They combined images captured under both front and back lighting, using the data to classify the leaf discs into three classes—resistant, moderate, and susceptible—based on the severity of the infection.

**Fig. 2:**
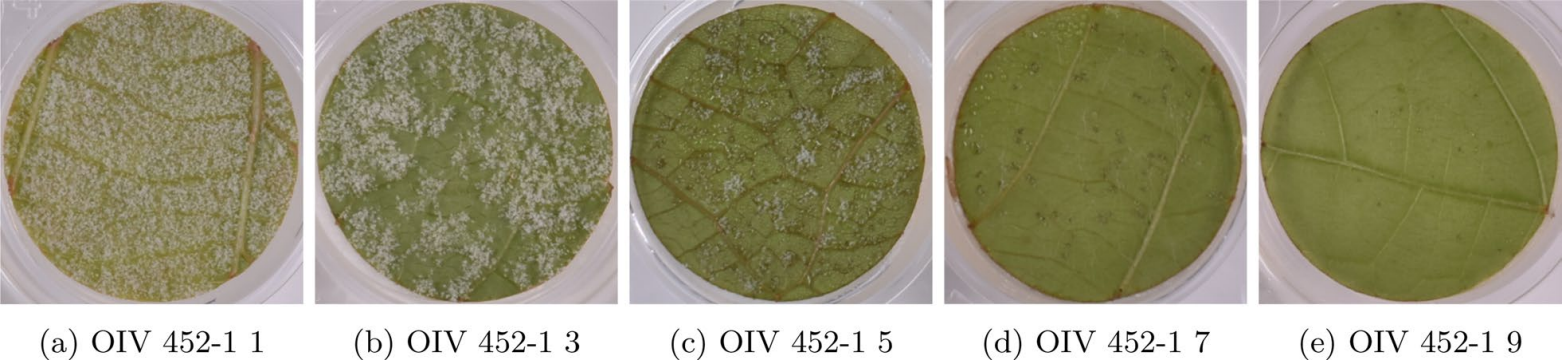
Grapevine leaf discs infected with *Plasmopara viticola* scored according to the OIV 452–1 scale. Score levels increase with resistance to the pathogen from 1 (very susceptible) to 9 (totally resistant). Level 1 (**a**) shows abundant sporulation densely covering the whole disc area, level 3 (**b**) shows abundant sporulation present in large patches, absence of plant necrosis, level 5 (**c**) shows limited sporulation present in intercostal patches, necrotic flecks, level 7 (**d**) shows sparse sporulation, necrotic spots and level 9 (**e**) shows no sporulation, possible presence of necrotic spots

In our approach, rather than just predicting a percentage of the sporulation surface, we used a neural network to predict a score in the OIV 452–1 [26] scale that takes into account both sporulation and different types of necrosis [27]. The OIV 452–1 is an ordinal scale used to assess the resistance of grapevines to downy mildew using leaf discs under controlled conditions. The scale has only odd values ranging from 1 (very susceptible) to 9 (totally resistant), with intermediate scores of 3 (less susceptible), 5 (partially resistant), and 7 (highly resistant). A description of the scale levels can be found in Fig. 2. A scale that considers both pathogen aggressiveness (sporulation) and the plant’s response (necrosis) provides a more accurate depiction of the interaction between the plant and the pathogen.

Compared to the meticulous task of directly annotating sporulation on leaf discs, the OIV 452–1 scale offers a more efficient and expedient method. Using a five-level scale with clearly defined categories, the OIV 452–1 simplifies the annotation process. The availability of this scoring scale has enabled experts to develop a substantial dataset containing thousands of annotated images, a considerable increase from the mere tens or hundreds typically used in previous studies. This wealth of data will allow the training of more sophisticated machine learning models, reducing the risk of overfitting.

## Methods

The four main steps of the proposed method are as follows: (i) Grapevine leaf discs were infected with *P. viticola* and placed in 12-well plates. (ii) An imaging system is used to capture images of the plates. (iii) The plate images form the basis of an OIV 452–1 scoring dataset. (iv) The dataset undergoes a preprocessing stage. (v) Various methodologies including convolutional neural networks and transformers have been explored for accurate analysis of the interactions between the plants pathogens.

### Leaf discs

#### Plant material

Between 2020 and 2023, multiple experiments were conducted to evaluate the resistance of various genotypes from different *Vitis* species, as well as breeding populations segregating for different *Rpv* loci, against *P. viticola*. Briefly, grafted plants were grown in 4 L pots in a greenhouse on a substrate composed of 1/3 perlite and 2/3 sand. Plants were watered daily with a complete nutritive solution (5% Plant Prod 17-10-20, Fertil SAS, France; 5% Plant Prod 20–20-20, Fertil SAS, France; 1.3% Yara Tera R, KRISTA MAG, Yara, France; and 0.005% PlantainFer, Plantin SARL, France).

#### Leaf disc infection

**Fig. 3:**
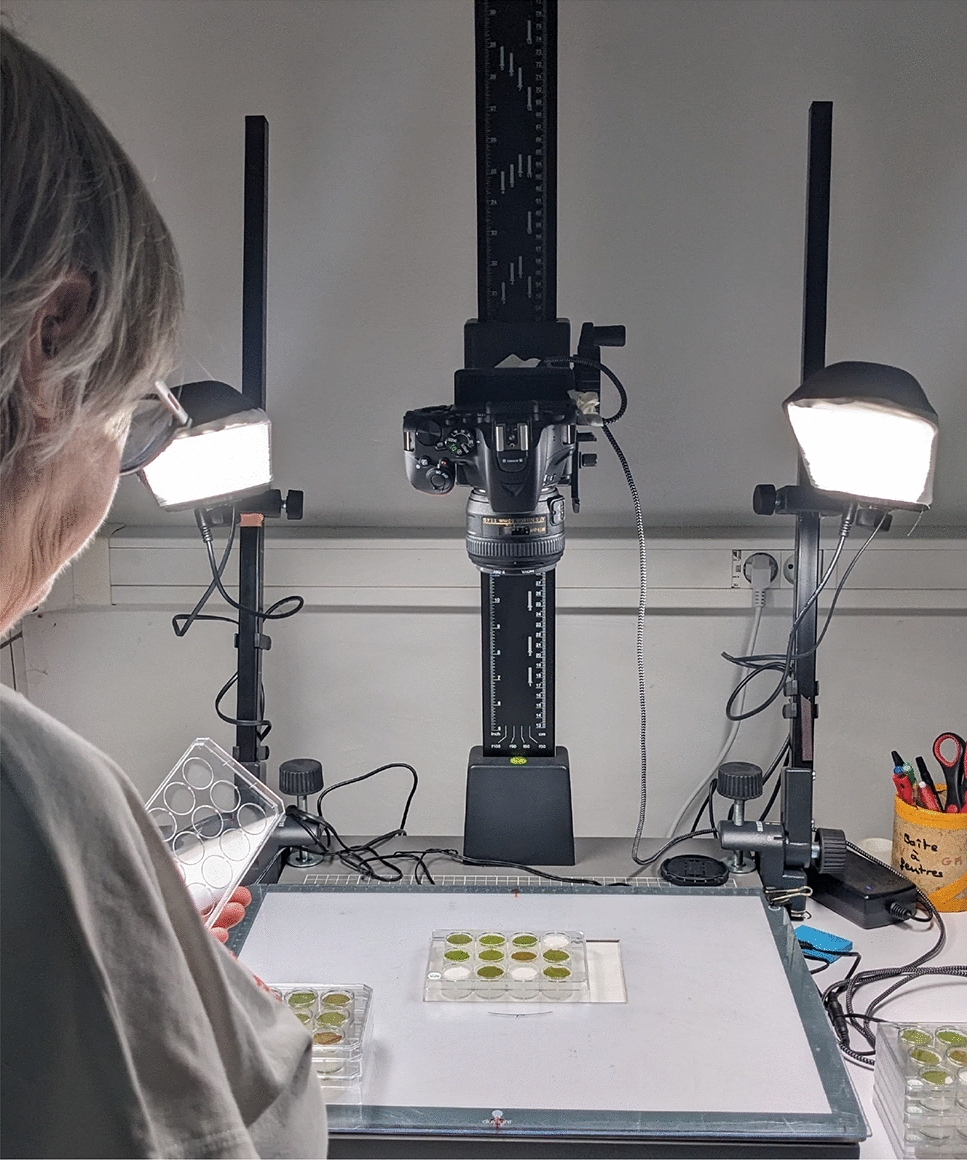
Imaging system used to produce the original plate images

A strain of *P. viticola* collected from *V. vinifera* variety Chardonnay in an experimental vineyard at INRAE-Colmar (France) in 2006 was maintained and propagated through spray inoculations on 6-week-old *V. vinifera * variety Muscat Ottonel, seedlings, which were subsequently placed in an open cardboard box covered with a plastic bag. For each bioassay, *P. viticola* sporangia were recovered from infected seedlings after 6 days of incubation in a growth chamber (21^∘^C, high relative humidity, 50 $\upmu$mol/m2/s light intensity) by leaf immersion in water and gentle shaking. For each plant growing in the greenhouse, at least three leaf discs (2 cm diameter) were sampled from the fourth and fifth fully expanded leaves from the grape shoot apex at the 10-leaf stage. The discs were placed on 12-well plates, on wet paper discs on agar solution (10 g/L), abaxial side up, and then artificially inoculated by spraying the *P. viticola* suspension (5x10^4^ sporangia/ml). The plates were then sealed and incubated in a growth chamber at 21^∘^C with a photoperiod of 14 h of light. After 24 h the innoculated droplets had disappeared. Inoculated leaf discs were evaluated between 3 and 6 days postinoculation using a stereo-microscope, with scores assigned according to the ordinal OIV 452–1 scale, according to [27] and described in Fig. 2. At the same time, leaf discs were also photographed using the setup shown in Fig. 3 and described below. Images, OIV 452–1 scores, and metadata for each leaf disc were stored in an Excel file database.

### Dataset created

The imaging system depicted in Fig. 3, features a fixed top-view RGB camera mounted on a copy stand. Two LCD lamps, on either side of the camera provide a homogeneous illumination. The RGB camera was a Nikon D5600, an APS-C 24.2 Mpx camera with a Nikkor 50 mm f1.4 lens, 75 mm equivalent to a 35 mm format. The camera was configured to capture images at a 6 Mpx resolution in JPEG format. Using this system, we generated 5193 plate images, encompassing 57,836 leaf discs. These discs must first be individually extracted from the images before they are analyzed for sporulation and necrosis. The subsequent sections explain this process in detail.

### Plate image preprocessing

**Fig. 4:**
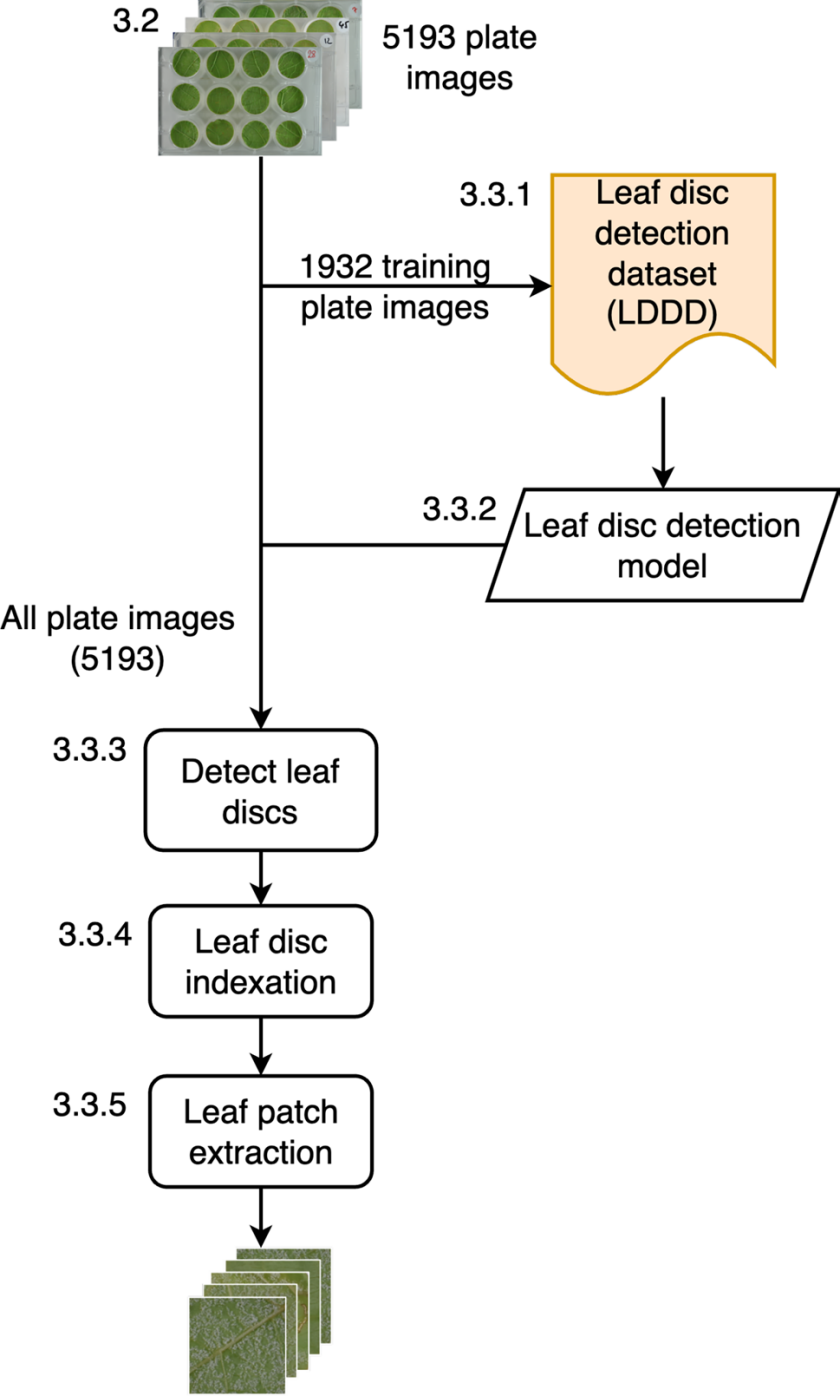
Preprocessing pipeline. The numbers in the figure and the caption correspond to the subsections of the manuscript. A total of 5193 plate images were produced (3.2). A subset of 1932 of these images was annotated to create a dataset with the position and size of leaf discs (3.3.1), which was then used to train a model to detect leaf discs (3.3.2). This model allowed the detection of leaf discs on all the available images (3.3.3). The leaf discs were indexed (3.3.4), and finally, the leaf patches were extracted (3.3.5)

Starting from the original plate images, which contained up to 12 leaf discs, a preprocessing pipeline was developed to automatically extract and index individual leaf patches from each plate image, as illustrated in Fig. 4. Indexing was essential for associating the extracted leaf patches with the experimental data and metadata.

#### Leaf disc detection dataset

To train the leaf disc detector model, the initial step of our preprocessing pipeline, we created a leaf disc detection dataset. Instead of manually annotating all available plate images, we sampled 1932 plate images from the 5193 available images, ensuring that each experiment from every year was represented. This dataset ultimately included bounding boxes for 22,122 leaf discs (fewer than the anticipated 23,184, due to some plates having fewer than 12 leaf discs), which were used as training, validation and test datasets. The plate images were split using stratification [28] on the year of the experiment, ensuring that images under each lighting condition were proportionally represented in the datasets. Among the leaf disc images, 15,530 were used for training, 3309 were used for validation, and the remaining 3273 were used for testing. This balanced dataset was used to train the object detector model.

#### Leaf disc detection model

We selected a Fast R-CNN model [29], from the PyTorch library [30], that was pretrained on COCO V1 [31]. We then selected a batch size of 20, the minimization was carried out using the ADAM optimizer [32] with a learning rate of $7\times 10^{-5}$. We performed 15 training sessions with a fixed learning rate and 15 more with learning rate decay using StepLR with a step size of 10 and a gamma of 0.8. Data augmentation was performed with the albumentations library [33] to increase data variability by changing the exposure with a random gamma transformation with a lower bound of 0.6 and an upper bound of 1.8 and using horizontal, vertical flipping and rotation. The best results were obtained with the model trained with StepLR, which had a mean loss of 0.016 and a standard deviation of 0.003, and the best model had a loss of 0.012.

#### Leaf disc detection

The best leaf disc detection model trained in the previous section was then used to detect the leaf discs in all 5193 available plate images and detect 57,836 leaf discs.

#### Leaf disc indexation

Since some plates might have missing columns of leaf discs, the algorithm could mistakenly interpret the empty space caused by the plate being positioned on the right side of the image as a missing column. To mitigate this, the first step of indexing the leaf discs was using a Hough line detector [34] to identify the left boundary of the plate. Two mean shift clustering [35] steps were then applied: one to assign the columns and the other to assign the rows. Finally, gaps between columns were analyzed to detect accurately any missing columns.

#### Leaf patch extraction

In [36], neural networks were used to automatically detect plant disease symptoms on detached leaves. The authors used an attention map visualization technique [37] to examine the residual errors, revealing that the neural networks could be sensitive to the background when making predictions. To avoid this issue, we extracted the largest square possible within the leaf disc, covering approximately 78% of its total area. These square images, henceforth referred to as “leaf patches” allow for a more focused analysis without background interference. One may wonder about the impact of this cropping. The border of leaf discs is considered a wound tissue offering artificial entrance for *P. viticola* zoospores (normal entrance takes place through stomata). It is known that defense mechanisms are weaker in the wounded border than in the rest of the leaf discs, allowing possible sporulation. The phenotype at the border is rarely from what is observed on the rest of the leaf disc: sporulation on the border versus absence of sporulation elsewhere. However, from a pure image processing point of view, if one were interested in the full leaf disc, it would be possible to transform the disc into a square via a cylinder to Cartesian representation. Our proposed approach could then be applied to these transformed images.

### OIV 452–1 scoring dataset

**Fig. 5:**
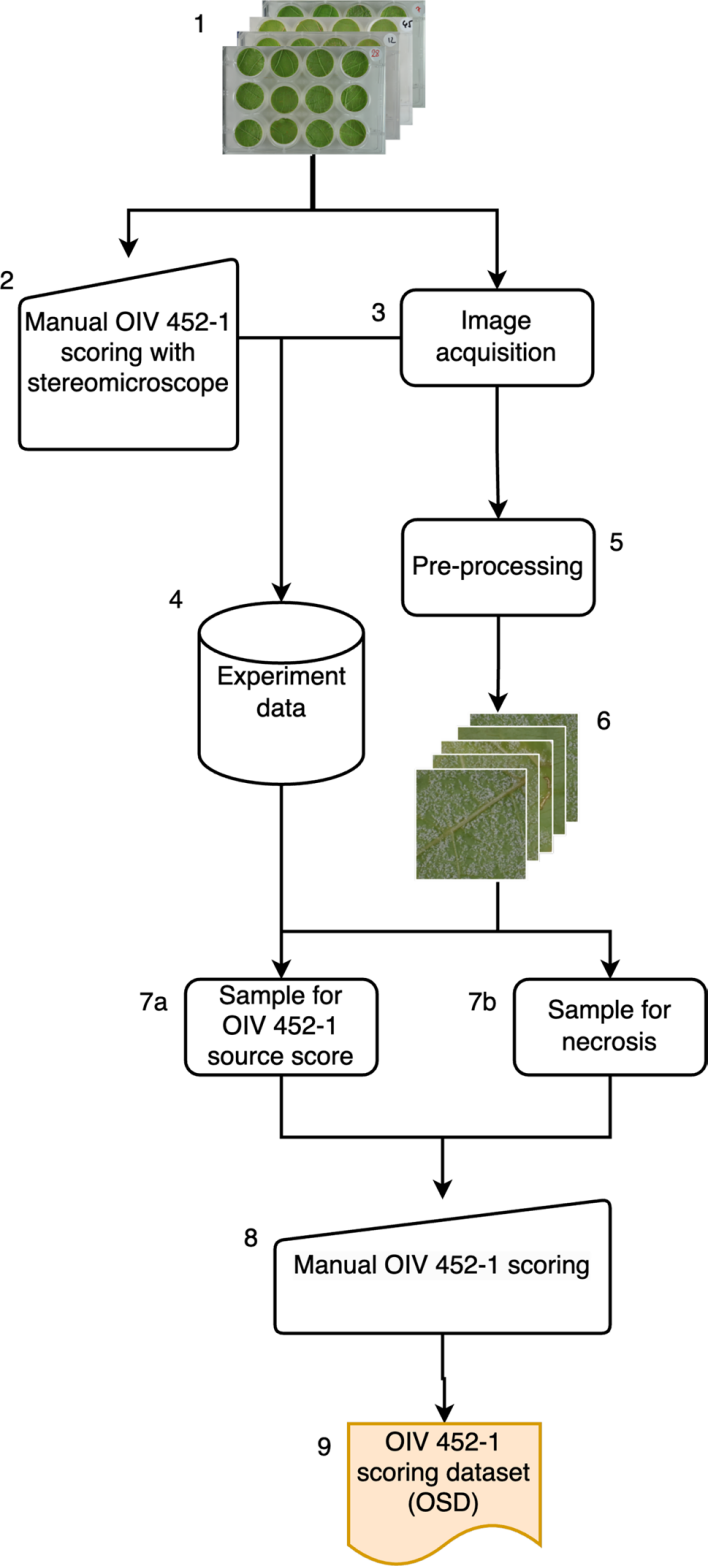
Setup of the OIV 452–1 scoring dataset. Plate images (3) were preprocessed to extract leaf patches (5), and the available leaf patches (6) were sampled to obtain images with all the symptoms described in Fig. 2 (7a, b). Experts manually scored the resulting patches with OIV 542–1 values (8) to obtain the OIV 452–1 scoring dataset (9)

**Fig. 6:**
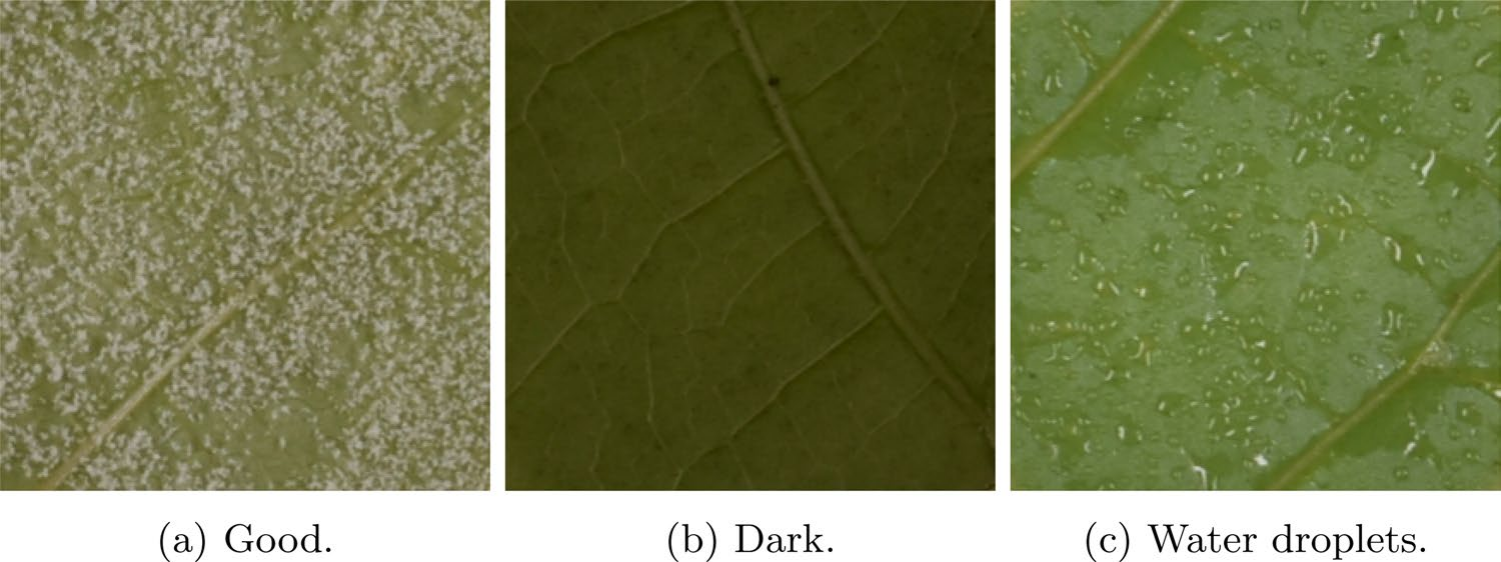
Examples of different issues present in the source images. Image (**a**) shows an example of an image labeled as “good”, without visible defects. Images with low reflectance such as (**b**) are labeled as “dark image” and can be fixed with preprocessing. The last image, (**c**), is labeled as “water droplets” as it contains water droplets that can in some cases be mistaken for sporulation

A flowchart of the pipeline used to create the OIV 452–1 scoring dataset is shown in Fig. 5. To create this dataset, we used the leaf patches created in section "Plate image preprocessing" that we sampled using two methods. First, we sampled images with low brightness and low hue values to improve the chances of selecting leaf patches containing various types of necrosis. Then, we used the existing database to sample images scored with all 5 levels of OIV 452–1 values. Two experts scored each leaf patch with the OIV 452–1 scoring scale using a computer interface specifically designed for this task A. Some images displayed artifacts such as low reflectance, resulting in dark images, or the presence of water droplets, as illustrated in Fig. 6. Given the prevalence of these issues, the model was designed to handle them effectively. The quality of the leaf patch images was systematically annotated, as detailed in Table 1.

**Table 1:** Distribution of image quality and OIV 452–1 scores

OIV 452-1	Dark	Good	Water droplets	Total
1	10	626	116	752
3	7	472	143	622
5	5	408	141	554
7	6	548	141	695
9	12	500	314	826

The samples of each type can be found in Fig. 6. “Dark” leaf patches come from underexposed images, “water droplets” are due to condensation and may look similar to sporulation, and “good” leaf patches correspond to well-exposed images without water droplets

The images were divided using stratification [28] on the experiment. The resulting dataset, summarized in Table 1, consists of 3449 images and was partitioned into training (2414 images), validation (517 images) and testing (518 images) sets. Stratification was used to ensure that OIV 452–1 scores and experiments were proportionally represented in each dataset.

### Deep learning methods

The OIV 452–1 scale is an ordinal discrete scale. We trained three different neural networks, using two different architectures, that could include this prior knowledge. We compared two competitive architectures: convolutional neural networks, which have been considered the state-of-the-art since their introduction, and transformer architecture, which have recently gained prominence in image processing [38]. To take into account the ordinality property we used the rank-consistent ordinal regression framework [39] that bridges the gap between classification and regression.

#### Rank-consistent ordinal regression based on conditional probabilities

Here we used rank-consistent ordinal regression based on conditional probabilities [39] (CORN). Let $D=\{x^{[i]}, y^{[i]}\}^{N}_{i=1}$ be a training set containing *N* samples where $x^{i}\in X$ denotes the inputs of the $i{-}th$ training sample and $y^{i}$ is its corresponding rank $y^{i}\in Y = \{r_1, r_2,..., r_k\}$, where the rank order is $r_k>r_{k-1}>...>r_1$. The objective of an ordinal regression model is to find a mapping $h:X\rightarrow Y$ that minimizes a loss (*h*). When using the CORN method, the last layer of the neural network contains $K-1$ neurons, representing binary tasks, and the output of each one is

1 $$\begin{aligned} f(x^{[i]}) = \hat{P}(y^{[i]}> r_{k}|y^{[i]} > r_{k-1}) \end{aligned}$$

the conditional probability of sample $x^{[i]}$ being of rank higher than *k*. By applying the chain rule, we can calculate unconditional probabilities such as

2 $$\begin{aligned} \hat{P}(y^{[i]} > r_{k}) = \prod _{j=1}^{k}f_{j}(x^{[i]}) \end{aligned}$$

and since $f_{j}(x^{[i]})$ is a probability, $\forall j, f_{j}(x^{[i]}) < 1$ from this, we can deduce that

3 $$\begin{aligned} \hat{P}(y^{[i]}> r_{1})>\hat{P}(y^{[i]}> r_{2})> \hat{P}(y^{[i]} > r_{K-1}) \end{aligned}$$

which proves that rank consistency is maintained. Finally, to calculate the value $\hat{y}^{i}$ corresponding to $x^{i}$, we predict the probabilities for each rank and sum the binary labels over a predefined threshold

4 $$\begin{aligned} \hat{y}^{i} = \sum _{j=1}^{K-1}\mathbb {1}(\hat{P}(y^{[i]}> r_{j}) > 0.5).\ \end{aligned}$$

#### Proposed network

**Fig. 7:**
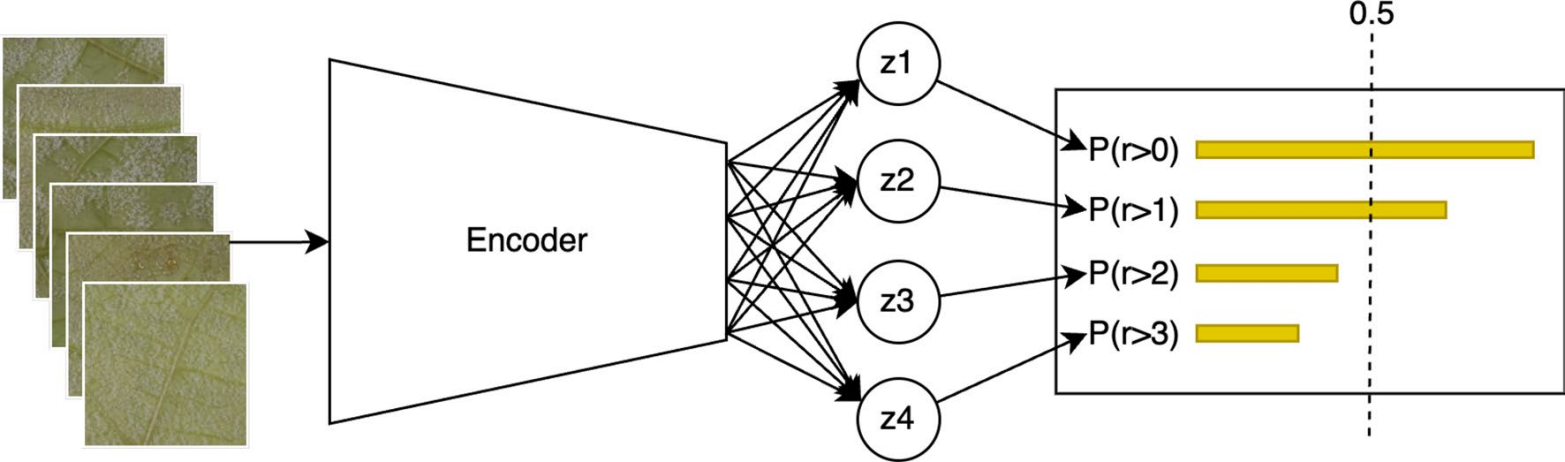
Rank-consistent ordinal regression (CORN) with a transformer encoder, shown example predicts an OIV 452–1 value of 5. After the encoder four neurons are responsible for predicting if the sample is of a rank higher than their index, for example, the neuron *z*1 predicts that a sample is of rank higher than 0 if its value is higher than the threshold 0.5. The rank of the sample corresponds to the sum of neurons with a value higher than the threshold. In this example, the sample has a rank of 2 (the rank 0 of the OIV 452–1 scale is 1 and the fourth rank is 9) that corresponds to an OIV 452–1 level 5

We used a Swin transformer [40] pretrained on ImageNet-1K [41] as the encoder. Figure 7 shows the full neural network. For the calculation to predict OIV 452–1 from rank prediction, a modification of Eq. 4 is used

5 $$\begin{aligned} OIV 452-1 = \left( \sum _{j=1}^{K-1}\mathbb {1}(\hat{P}(y^{[i]}> r_{j}) > 0.5)\right) *2+ 1.\ \end{aligned}$$

We also tested two convolutional neural network architectures, ResNet50 [42] and ConvNeXT [43] both also pretrained on ImageNet-1K. All pretrained backbones were selected from the Hugging Face’s [44] model hub.

#### Training

Leaf patch preprocessing was performed before the images were sent to the neural network. First a linear transformation was applied to fix underexposed images to diminish lightning heterogeneity. Afterward, image augmentation was performed using the albumentations [33] library to increase the number of available samples. The selected random augmentations were rotations, vertical and horizontal symmetries, brightness and contrast modifications with brightness and contrast limits of 0.15 and 0.25, respectively, and random gamma with upper and lower bounds of 0.6 and 1.2, respectively. We selected a batch size of 771 to fill 80% of the GPU, an NVIDIA A100 80 G, and a learning rate of $3625\times 10^{-4}$. The models were trained for a maximum of 200 epochs with an early stopping monitoring mean average error and a waiting time of 15 epochs. Each model was trained 15 times.

#### Model metrics

We compared the performances of three encoders—Swin transformer, ResNet50 and ConvNeXT—using three different metrics. The mean absolute error (MAE)

6 $$\begin{aligned} MAE = \frac{\sum _{i=1}^{N}|y_i-\hat{y}_i|}{N} \in [0,1], \end{aligned}$$

that calculates the average distance between the ground truth and the prediction. The mean square error (MSE)

7 $$\begin{aligned} MSE = \frac{\sum _{i=1}^{N}(y_i-\hat{y}_i)^2}{N} \in [0,1] \end{aligned}$$

which also calculates the distance between the ground truth and prediction, but in this case, it inflicts a penalty that increases with the difference between the ground truth and the prediction and accuracy

8 $$\begin{aligned} accuracy = \frac{TP+TN}{TP+TN+FP+FN} \in [0,1], \end{aligned}$$

a ratio of the correct predictions over the sum of predictions. TP, TN, FP and FN represent true positives, true negatives, false positives, and false negatives, respectively. The mean absolute error and mean square error best values are close to 0. The best accuracy values are those close to 1. Finally, we use

9 $$\begin{aligned} F1-score = \frac{2*TP}{2*TP+FP+FN} \in [0,1] \end{aligned}$$

to analyze the model’s performance on each separate label.

### Differentiation of genotypes with different levels of resistance

To evaluate the model’s ability to differentiate genotypes with varying resistance levels, we designed an experiment using images of biological material not included in the training datasets.

To achieve this goal, we created a genotype differentiation dataset. We selected nine genotypes from the progeny of a cross between two resistant parents, representing varied resistance levels to *P. viticola* across the OIV 451–1 scale (see section "Plant material" for plant material production). In 2023, three experiments were conducted using between 3 and 5 leaf discs per genotype, yielding 114 leaf discs. These discs were manually scored by an expert and photographed at 0, 3, 4, 5, and 6 days postinoculation (dpi). The resulting dataset contained 570 observations, each containing the identifier, genotype, dpi, and OIV 452–1 score for each leaf disc.

To compare the model’s performance with human scoring across the set of nine contrasting genotypes, we first predicted the OIV 452–1 values for all observations. An ANOVA test was then conducted, and we compared the F-scores. Additionally, a Tukey honest significant difference test was employed to evaluate the similarity between the results of both methods.

## Results and discussion

### OIV 452–1 prediction

First, we compared the performances of the three selected encoders: the Swin transformer, ResNet50 and ConvNeXT. We trained neural networks with the three encoders 15 times each and the results can be seen in Table 2. Swin transformers, slightly but systematically, outperformed convolutional neural network-based architectures in terms of all the metrics. For the remainder of the work, we retained the best network using the Swin transformer encoder.

**Table 2:** OIV 452–1 ordinal regression performance per encoder with standard deviation

Encoder	MAE (Eq. 6)	MSE (Eq. 7)	Accuracy (Eq. 8)
Swin Transformer	0.188±0.006	0.206±0.015	0.817±0.006
ConvNeXT	0.195±0.009	0.217±0.011	0.811±0.011
ResNet50	0.215±0.011	0.242±0.015	0.793±0.011

Next, we selected the best model among those that had a Swin transformer as the encoder, and we selected the model with the lowest MSE value, which was 0.203.

In addition to global metrics such as the MAE, MSE, and accuracy, we examined the confusion matrix, as shown in Table 3. Notably, the model never deviated by more than one rank on the OIV 452–1 scale. This indicates that the model performs well, primarily struggling with ambiguities between consecutive classes. Upon further investigation of the residual errors, we observed that water droplets, such as those shown in Fig. 6c, were sometimes mistaken for sporangia by the model. Additionally, an abundance of water droplets hindered the detection of sporangia beneath them. The impact of these droplets varies across OIV 452–1 levels. At low resistance levels, such as values of 1 and 3, the presence of artifacts resembling sporulation had minimal impact on the predictions. However, at higher levels of resistance such as 7 or 9, a single water droplet misidentified as sporangia can alter the classification of the leaf patch.

**Table 3:** Confusion matrix and F1-score of the test dataset after when using the best model

True OIV 452-1	Predicted OIV 452-1						F1-Score (Eq. 9)
	1	3	5	7	9		
**1**	109	4	0	0	0	**1**	0.93
**3**	13	68	12	0	0	**3**	0.76
**5**	0	15	63	5	0	**5**	0.75
**7**	0	0	9	76	19	**7**	0.73
**9**	0	0	0	28	96	**9**	0.77

To better understand the impact of water droplets on model performance, we divided the test dataset into two subsets: one containing clear images and the other containing images with water droplets. The MSE values for the clear and the water-droplet images were 0.193 and 0.236, respectively (data not shown), indicating a decline in performance when predicting leaf patches that contain water droplets.

### Differentiation of genotypes with different levels of resistance

As an additional evaluation of our model, we conducted a statistical comparison between the model’s predictions on leaf patches and human assessments using a stereo-microscope on whole leaf discs.

**Fig. 8:**
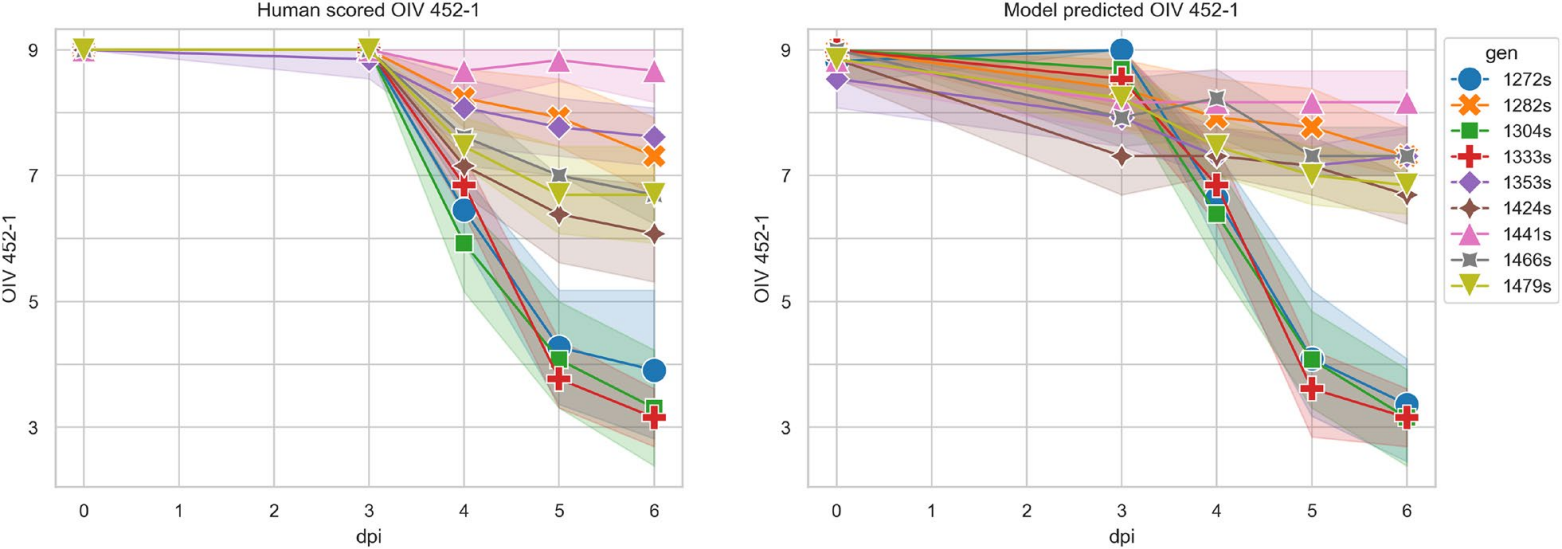
Disease evolution over time (during 6 days) of the disease scored by OIV 452–1 values by humans (left) and predicted by the model (right) of 9 genotypes with various levels of resistance with error bands. Colors represent genotypes, the symbols represent the average for each genotype at each time point

Figure 8 illustrates the progression of the disease over 6 days across the 114 leaf discs from the genotype differentiation dataset; cf. Figure 1. The left panel shows the human assessment, while the right panel shows the model’s predictions. The most significant discrepancy between the two annotations appeared at 3 dpi, when human scoring did not detect genotype differences, yet the model predictions did. This disparity is likely due to condensation-induced water droplets, which are the most prevalent on the leaf discs at this stage. As shown in Fig. 8, the sixth day after infection showed the greatest divergence between genotypes.

To compare the model and the human annotations, we performed an ANOVA with data from the last day (6 dpi) for both human scoring and model predictions. This analysis aimed to determine to what extent the OIV 452–1 score could be explained by the genotype. Table 4a, b show the ANOVA results for human scoring and predicted values, respectively. These two tables show p values and F scores for both manual and automatic annotation for predicting the effect of genotypes on OIV 452–1 values. It is worth noting that the model exhibited a greater F value when distinguishing between genotypes. Both ANOVA analyses revealed no significant interaction between genotypes and experiments, enabling us to use a Tukey honestly significant difference test to verify whether the model and manual scores yielded consistent results when comparing genotypes pairwise.

**Table 4:** ANOVA Tables for both scored (Table a) and predicted (Table b)

	Sum of squares	Degrees of freedom	F	p-value
(a) ANOVA for scored data.				
Genotype	400.38	8	35.03	4.40e-24
Experiment	27.64	2	9.68	1.60e-04
Interaction	32.44	16	1.42	1.52e-01
Residual	124.27	87		

	Sum of squares	Degrees of freedom	F	p-value
(b) ANOVA for predicted data.				
Genotype	425.37	8	58.50	9.33e-32
Experiment	19.02	2	10.47	8.46e-05
Interaction	14.28	16	0.98	4.83e-01
Residual	79.07	87		

The Tukey honestly significant difference test resuklts are as follows: out of 36 possible pairwise comparisons, the model and manual scoring agree on 35, yielding an accuracy score of 0.97. In most cases, when both methods reject the null hypothesis, the model provides stronger p values. Only one genotype pair 1441s and 1466s, produced differing results between the two methods. As presented in Table 5 for genotype 1441s, the model’s mean prediction was 8.17 instead of 8.67, with three images predicted as OIV 452–1 score 7 instead of 9. Inspection of these three images revealed white artifacts that were indistinguishable from sporulation due to their low resolution. In contrast, genotype 1466s had a mean model prediction of 7.31 instead of 6.69, which was attributable to two factors. First, two leaf patches were predicted to have an OIV 452–1 score of 7 instead of 5, these patches were at the borderline between these two ranks. Second, two leaf patches were predicted to have OIV 452–1 scores of 9 instead of 7, although no sporulation was visible on either the leaf patches or the full leaf disc images. It is hypothesed that the low resolution of the leaf patch images obscured sporulation.

**Table 5:** Average OIV 452–1 and standard deviation for each genotype in the genotype differentiation dataset as scored by humans and predicted by the model

Genotype	OIV 452-1	
	Human scored	Model predicted
1272s	3.90±2.07	3.36±1.50
1282s	7.30±1.10	7.30±0.75
1304s	3.30±1.79	3.15±1.51
1333s	3.15±0.98	3.15±0.98
1353s	7.61±0.96	7.30±0.75
1424s	6.07±1.55	6.69±0.75
1441s	8.66±0.77	8.16±1.02
1466s	6.69±0.75	7.30±0.75
1479s	6.69±1.37	6.84±0.98

These results can now be explored further in multiple directions. Improving the resolution of the plate images and reducing water droplets on the leaf discs during acquisitions could enhance the model’s performance. This precise and efficient deep learning method could replace visual observation when assessing symptoms, opening up new avenues for investigations such as identifying new *Rpvs* and studying *P.viticola* strains/*Rpv* interactions. All leaf discs used in this work were maintained under controlled conditions without exposure to other pathogens. Sampling leaf discs from naturally infected grapevine plants grown in vineyards would be valuable for evaluating model performance under new conditions and determining whether other pathogens affect model performance. This approach could be a stepping stone toward noninvasive diagnosis in vineyards. Finally, the application of this pipeline could be expanded in two ways. First, a tool such as MANINI [45] could be employed to adapt the model for images from other platforms using the same pathosystem. Second, this approach could be extended to other plant—pathogen interactions, such as grapevine and powdery mildew or black rot, since both of these pathosystems have an associated OIV scale.

## Conclusions

Downy mildew caused by *Plasmopara viticola* is one of the most destructive diseases affecting grapevines. Breeding-resistant varieties require high-throughput and precise phenotyping methods to evaluate resistance. Grapevine resistance is primarily characterized by either the absence or reduction of sporulation, along with potential necrosis. The key challenge lies in developing a phenotyping tool capable of accurately analyzing both variables.

In this paper, we introduced a comprehensive image processing and machine learning pipeline to analyze the interaction between grapevine leaf patches and downy mildew using existing images. We employed the standard ordinal scale OIV452–1, which accounts for both sporulation and necrosis, to score symptoms on leaf discs. The pipeline fully leverages existing images to characterize these two symptoms.

Various strategies were evaluated to incorporate the ordinal scale’s prior knowledge, which reflects the progression of sporulation and necrosis throughout the infection. The best ordinal regression performance was achieved using a Swin transformer as the encoder, with this neural network reaching an MSE of 0.203 on the test dataset and ensuring that the discrepancy between the predicted OIV 452–1 and the ground truth never exceeded one class. Additionally, the model demonstrated a significant advantage. While a standard scoring session typically takes 4 h for a human to assess up to 2000 discs, the model only needed 22 s to score the same number using our setup.

This method successfully differentiates genotypes across a wide range of phenotypes. By comparing the model’s predictions on leaf patch images to human assessments on full leaf discs using a stereo-microscope, we achieved an accuracy of 0.97 using the Tukey honestly significant difference test with data from both sources. In conclusion, the model effectively captured the biological information of the pathosystem with precision and aligned closely with human observations.

## Figures and Tables

**Fig. 9:**
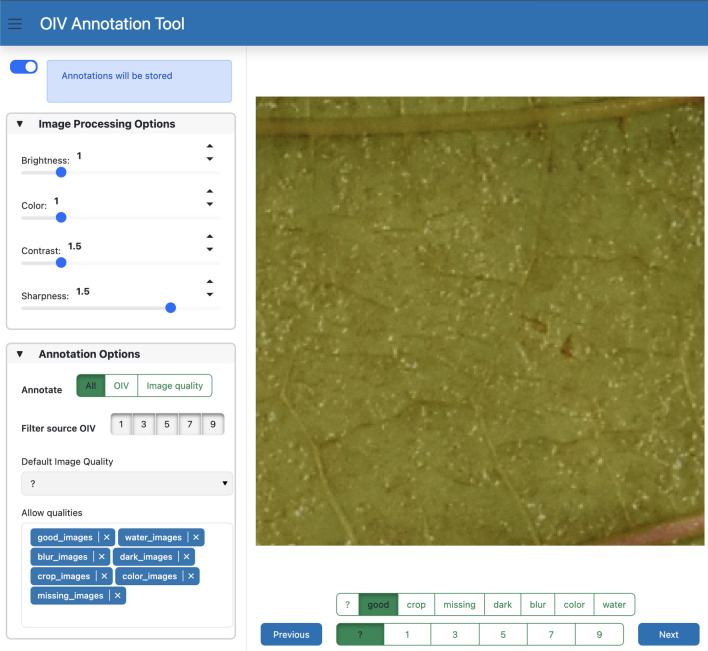
Annotation user interface developed to score leaf patches with OIV 452–1 values

## Data Availability

The data, models and computer user interface used for leaf patches annotations will be available after acceptance upon reasonable request.

## Appendix A Annotation user interface

To annotate leaf patches with OIV 452–1 scores and quality data, we created the user interface shown in Fig. 9. To enable the annotation process, the tool allows limited image manipulation. Upon acceptance of the manuscript, the tool will be available at https://huggingface.co/treizh/oiv_ld_phenotyping.
